# High-oleate yeast oil without polyunsaturated fatty acids

**DOI:** 10.1186/s13068-018-1131-y

**Published:** 2018-05-09

**Authors:** Vasiliki Tsakraklides, Annapurna Kamineni, Andrew L. Consiglio, Kyle MacEwen, Jonathan Friedlander, Hannah G. Blitzblau, Maureen A. Hamilton, Donald V. Crabtree, Austin Su, Jonathan Afshar, John E. Sullivan, W. Greg LaTouf, Colin R. South, Emily H. Greenhagen, A. Joe Shaw, Elena E. Brevnova

**Affiliations:** 1 Novogy, Inc, 85 Bolton Street, Cambridge, MA 02140 USA; 2 Present Address: Ginkgo Bioworks, 27 Drydock Avenue, 8th Floor, Boston, MA 02210 USA

**Keywords:** Oleic acid, Oleate, Monounsaturated, Oil, Triglyceride, Triacylglyceride, TAG, Yeast, Lubricant, Oxidative stability

## Abstract

**Background:**

Oleate-enriched triacylglycerides are well-suited for lubricant applications that require high oxidative stability. Fatty acid carbon chain length and degree of desaturation are key determinants of triacylglyceride properties and the ability to manipulate fatty acid composition in living organisms is critical to developing a source of bio-based oil tailored to meet specific application requirements.

**Results:**

We sought to engineer the oleaginous yeast *Yarrowia lipolytica* for production of high-oleate triacylglyceride oil. We studied the effect of deletions and overexpressions in the fatty acid and triacylglyceride synthesis pathways to identify modifications that increase oleate levels. Oleic acid accumulation in triacylglycerides was promoted by exchanging the native ∆9 fatty acid desaturase and glycerol-3-phosphate acyltransferase with heterologous enzymes, as well as deletion of the Δ12 fatty acid desaturase and expression of a fatty acid elongase. By combining these engineering steps, we eliminated polyunsaturated fatty acids and created a *Y. lipolytica* strain that accumulates triglycerides with > 90% oleate content.

**Conclusions:**

High-oleate content and lack of polyunsaturates distinguish this triacylglyceride oil from plant and algal derived oils. Its composition renders the oil suitable for applications that require high oxidative stability and further demonstrates the potential of *Y. lipolytica* as a producer of tailored lipid profiles.

**Electronic supplementary material:**

The online version of this article (10.1186/s13068-018-1131-y) contains supplementary material, which is available to authorized users.

## Background

Oil is a vital facilitator of world economic growth with applications ranging from fuels, lubricants, plastics, and chemicals to food, pharmaceuticals, and personal-care products. The variety in applications arises from the wide range of properties exhibited by oils of different compositions. The majority of oil used in industry is derived from petroleum and its refined components with bio-based oil making up a small fraction [1]. Despite the great diversity of lipids made by living organisms, large scale quantities have been produced from a small number of agricultural crops and domesticated animals and are limited in composition and hence in applications. Biotechnology enables the manipulation of the lipid composition of oleaginous, culturable organisms such as certain yeast and algae [2], creating a flexible platform for the production of tailored oils with potentially improved or novel properties. Petrochemical substitutes can thus be produced at large scale on a variety of feedstocks to address specific oil applications.

Safe, genetically tractable, and industrially robust, *Yarrowia lipolytica* is becoming a preferred platform for metabolic engineering [3–6] to produce acetyl-CoA-derived molecules such as usual and unusual fatty acids [7–10], fatty alcohols [11], fatty esters [12], beta carotenoids [13], alkanes [14], and terpenes [15]. Lipid accumulation is induced by nutrient limitation in the presence of excess carbon and involves fatty acyl-CoA synthesis via a type I fatty acid synthase (FAS), modification of chain length and degree of desaturation by elongases and desaturases, and incorporation into triacylglyceride (TAG) via a series of enzymatic steps (reviewed in [16]): glycerol-3-phosphate acyltransferase (GPAT) attaches the first fatty acid onto the glycerol backbone to produce lysophosphatidic acid (LPA); lysophosphatidic acid acyltransferase (LPAT) attaches a second fatty acid to produce phosphatidic acid (PA); PA is dephosphorylated by phosphatidate phosphatase (PAP) to produce diacylglycerol (DAG); diacylglycerol acyltransferase (DGAT) activities add a final fatty acid to produce TAGs. The *Y. lipolytica* genome encodes two elongases (*YALI0F06754*, *YALI0B20196*), a Δ9 desaturase (*OLE1 YALI0C05951*), a Δ12 desaturase (*FAD2 YALI0B10153*), two GPATs (*SCT1 YALI0C00209*, *GPA YALI0A10362*), one LPAT (*SLC1 YALI0E18964*), one PAP (*PAH1 YALI0D27016*), and two DGATs (*DGA1 YALI0E32769*, *DGA2 YALI0D07986*) [17–19]. Pathway conservation between *Saccharomyces cerevisiae* [20, 21] and *Y. lipolytica* suggests that there is also significant crosstalk between the TAG biosynthesis pathway and phospholipid synthesis.

In this study, we explored the potential of *Y. lipolytica* as a high-oleate TAG oil producer. High-oleate TAG oil has applications in the food industry, as a chemical intermediate for oleic acid-derived products, and as a lubricant. Two key qualities of oil intended for lubrication are high oxidative stability and the ability to remain liquid at a wide range of operating temperatures [22]. Oxidative stability correlates with the number of double bonds in the fatty acid carbon chain. Polyunsaturated fatty acids are especially vulnerable to oxidative attack and oxidation is the primary mechanism of lubricant degradation. Saturated fatty acids have oxidative stability, but limited temperature range. Monounsaturated fatty acids are an excellent compromise combining good oxidative stability and a wide useful temperature range [23]. The native composition of *Y. lipolytica* TAG oil consists mainly of long chain fatty acids with up to two desaturated bonds (C16:0, C16:1 Δ9, C18:0, C18:1 Δ9, and C18:2 Δ9Δ12) [24]. We used gene deletion and expression of heterologous genes to tailor lipid synthesis and TAG accumulation for the production of biolubricant oil that has properties as good or better than petroleum-based oil. We report the engineering of a *Y. lipolytica* strain that produces oil highly enriched in monounsaturated and devoid of polyunsaturated fatty acids with an oleate content exceeding 90% of total fatty acids.

## Results and discussion

We previously identified wild-type *Y. lipolytica* strain YB-392 as a suitable starting strain based on minimal citric acid secretion, non-hyphal morphology and ease of genetic manipulation [24]. We studied the effects of a set of genetic modifications in fatty acid synthesis and TAG storage in this background and then combined those modifications favorable to oleate accumulation into a single strain.

### Elimination of polyunsaturated fatty acids

**Fig. 1:**
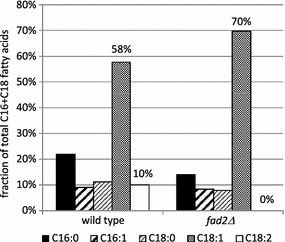
Deletion of the native Δ12 fatty acid desaturase leads to elimination of linoleic acid. Wild-type and *Δfad2* strains were grown in nitrogen-limited media for 96 h and cells were analyzed by gas chromatography to obtain the lipid profile shown

In *Y. lipolytica*, oleate (C18:1) is converted to linoleate (C18:2) through desaturation by the Δ12 fatty acid desaturase encoded by *FAD2 YALIOB10153* [18]. Deletion of this gene (*Δfad2*) leads to elimination of all detectable linoleic acid and a concomitant increase in oleic acid (Fig. 1).

### GPAT engineering

**Fig. 2:**
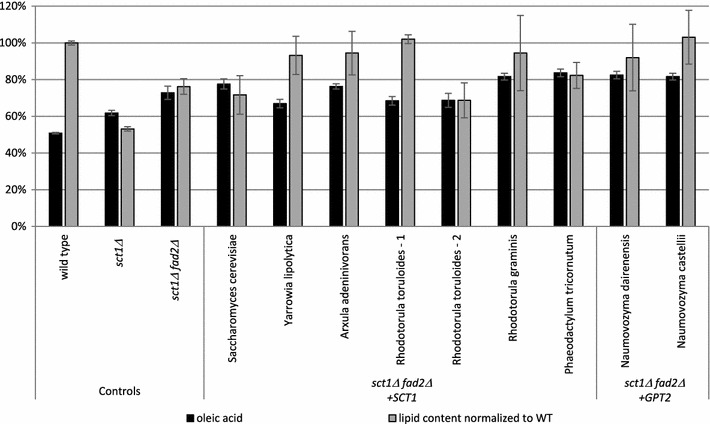
Heterologous GPAT expression in *Y. lipolytica*. 8 heterologous GPAT genes and the native GPAT *YlSCT1* were each transformed into a *Δsct1 Δfad2* double deletion strain. 16 transformants of each gene as well as duplicates of control strains were grown in nitrogen-limited media for 72 h. Cells were analyzed by fluorescence assay and gas chromatography and the average values across transformants are shown. Error bars represent standard deviation values. Total lipid content is reported as arbitrary units of fluorescence divided by optical density at 600 nm (FL/OD) normalized to the wild-type strain. Oleic acid levels are reported as the percent fraction of total C16 and C18 fatty acids quantified

In the course of studying the effect of lipid pathway gene deletion and overexpression, we found that the endogenous GPAT enzyme encoded by *YALI0C00209* (*YlSCT1*) has a strong preference for saturated fatty acids: deletion of *YlSCT1* leads to an increase in the oleate fraction and a reduction in the total lipid content of the cell (Fig. 2, compare *Δsct1* to wild-type). We tested a small library of GPAT-encoding genes to determine whether they could restore lipid production in the absence of native *YlSCT1* while maintaining the increased relative oleate content seen in this deletion mutant. The heterologous genes examined were *SCT1* homologues from *Saccharomyces cerevisiae, Arxula adeninivorans, Rhodotorula graminis*, *Phaeodactylum tricornutum* and 2 candidates from *Rhodotorula toruloides,* as well as homologues of the *Saccharomyces cerevisiae* GPAT gene *GPT2* from *Naumovozyma dairenensis* and *Naumovozyma castellii* (sequences provided in Additional file 1). These candidates share 39–52% primary sequence identity with YlSct1 protein. Expression cassettes containing *YlSCT1* or one of these 8 heterologous GPAT genes under the control of the strong constitutive *YlTEF1* promoter and coupled to a zeocin resistance marker were transformed into a *Δsct1 Δfad2* double deletion strain (Fig. 2). GPAT genes that did not improve lipid content were not investigated further. Failure to improve lipid content could be related to poor expression or poor enzyme activity in *Y. lipolytica* (e.g., misfolding, absence of post-translational modification, mislocalization, and low activity for native fatty acids). We found that several GPATs both increase lipid content to wild-type levels and maintain the high-oleate composition of the parent strain. These findings underline the importance of engineering acyltransferase specificity to tailor lipid content by stabilizing the desired fatty acids into storage lipids. *A. adeninivorans SCT1* (*AaSCT1*) was selected for subsequent strain engineering due to its positive effect on lipid content, maintenance of high-oleate levels and the close homology of this yeast with *Y. lipolytica* [25].

### Δ9 desaturase engineering

**Fig. 3:**
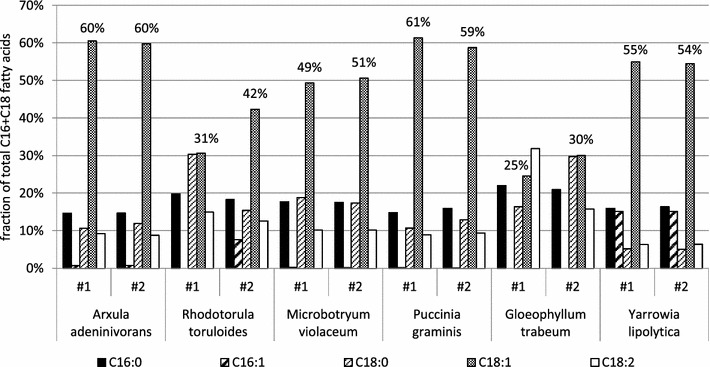
Heterologous Δ9 fatty acid desaturase expression in *Y. lipolytica*. Of the 7 heterologous Δ9 fatty acid desaturase genes individually transformed into a *Δole1* strain, 5 gave rise to transformants able to grow without monounsaturated fatty acid supplementation. Two isolates of each gene as well as duplicates of the wild-type strain were grown in nitrogen-limited media for 96 h and cells were analyzed by gas chromatography to obtain the lipid profile shown

*Y. lipolytica* expresses a single Δ9 fatty acid desaturase and produces two Δ9-desaturated fatty acids: palmitoleate (C16:1 Δ9) and oleate (C18:1 Δ9). We sought to alter the unsaturated lipid profile of our strain to decrease palmitoleate in favor of oleate. To this end, we deleted the native Δ9 fatty acid desaturase gene (*ole1::hph*), leading to auxotrophy for monounsaturated fatty acids. We confirmed that transformation of this strain with *YlOLE1* could rescue growth on unsupplemented media and we used this selection to investigate the lipid profile phenotypes of a set of heterologous Δ9 fatty acid desaturase genes (sequences provided in Additional file 1) targeted to replace the antibiotic-resistance marker at the *ole1::hph* locus. Transformation with two *OLE1* homologues from *Rhodotorula toruloides* and *Rhodotorula graminis* gave no viable colonies on unsupplemented media, suggesting that these genes did not successfully express a Δ9 fatty acid desaturase capable of complementing the endogenous desaturase deletion. As in the case of GPAT genes that did not yield the desired phenotype, these *OLE1* homologues were not investigated further. The lack of oleic acid prototrophy complementation could be due to poor expression or poor enzyme activity. On the contrary, genes from *Arxula adeninivorans*, *Microbotryum violaceum*, *Puccinia graminis*, *Gloeophyllum trabeum,* and a second homologue from *Rhodotorula toruloides* all complemented *Δole1* for growth on unsupplemented media. Two isolates of each transformation were chosen for further analysis. PCR analysis of the genomic locus as well as plating on hygromycin-containing media confirmed the insertion of the heterologous *OLE1* genes in place of the hygromycin resistance gene (data not shown). We compared the lipid profiles of the ten heterologous *OLE1* strains to wild-type (Fig. 3). The heterologous desaturases included in this study exhibited a strong preference for stearate (C18:0) over palmitate (C16:0) as the substrate of the desaturation reaction. *A. adeninivorans* and *P. graminis OLE1* resulted in higher relative oleate content than the native gene.

### High-oleate strain engineering and characterization

**Fig. 4:**
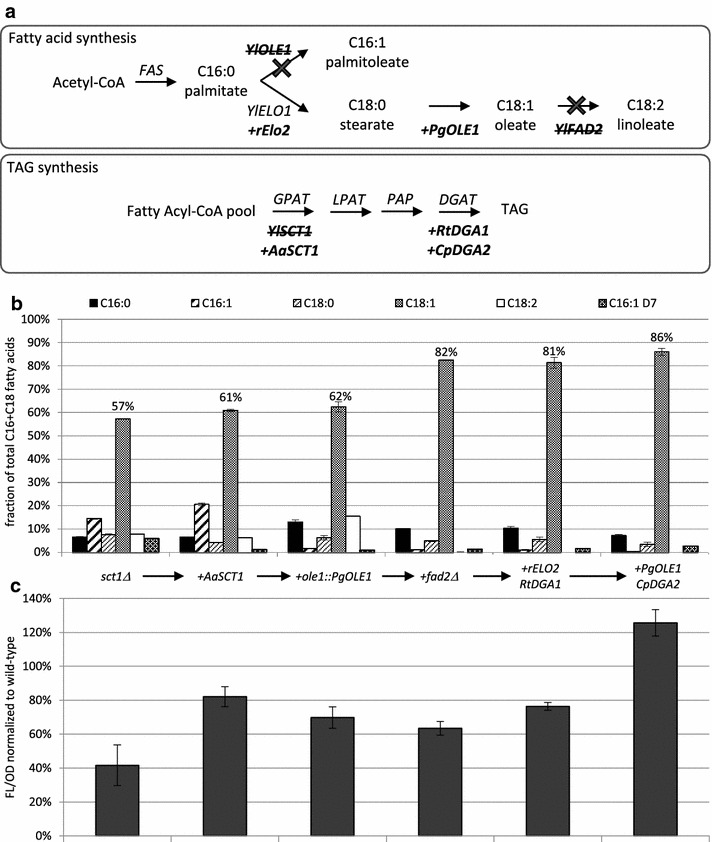
High-oleate strain engineering. A simplified pathway for fatty acid and TAG synthesis in *Y. lipolytica* is used to illustrate the gene deletions (*Ylole1*, *Ylsct1*, *Ylfad2*) and heterologous gene expressions (*PgOLE1*, *AaSCT1*, *rELO2*, *RtDGA1*, *CpDGA2*) incorporated into the high-oleate strain NS993 (**a**). The indicated strains were grown in triplicate in nitrogen-limited media for 96 h. Cells were analyzed by gas chromatography and fluorescence assay to obtain the lipid profile (**b**) and lipid content (**c**) shown. Fatty acid levels are reported as the percent fraction of total C16 and C18 fatty acids quantitated. Total lipid content is reported as FL/OD normalized to wild-type. Values shown are the average of triplicate cultures and error bars represent standard deviation. *FAS* fatty acid synthase, *GPAT*: glycerol-3-phosphate acyltransferase, *LPAT* lysophosphatidic acid acyltransferase, *PAP* phosphatidate phosphatase, *DGAT* diacylglycerol acyltransferase, *TAG* triacylglycerol

We combined the findings above to engineer a high-oleate strain (Fig. 4). First, a zeocin resistance cassette was used to express *AaSCT1* (*Y. lipolytica TEF1* promoter) in the *Δsct1* background resulting in an increase in oleate levels. We observed that *sct1*-deleted strains accumulate an oleate degradation product, 7-hexadecenoate C16:1 Δ7, and included this species in our lipid composition analysis from this point forward. This fatty acid, the result of a single round of β-oxidation of oleic acid, was previously observed in strains, where the glycerol-3-phosphate shuttle was disrupted through *gut2* deletion [26, 27]. We hypothesize that the remaining GPAT activity in *sct1*-deleted cells has a substrate preference for this degradation product and leads to its enrichment in TAG. C16:1 Δ7 levels decreased from 6 to 1% of total fatty acids with expression of *AaSCT1* (Fig. 4b, compare *Δsct1 AaSCT1* to *Δsct1*).

To shift monounsaturated content from C16 to C18, we then replaced the native Δ9 desaturase with *P. graminis OLE1* as before (Fig. 3). The resulting *Δsct1 AaSCT1 ole1::PgOLE1* strain exhibits the C18:0 substrate preference previously observed. Palmitoleic acid is reduced from 21% in the native *YlOLE1* parent strain to 2%, while C18 Δ9 products (oleate and linoleate) increase from 67 to 78% (Fig. 4b).

To eliminate polyunsaturated fatty acids, the Δ12 desaturase was next deleted. The effect of this modification was particularly pronounced due to the high linoleate content of the parental strain (Fig. 4b). No linoleate could be observed upon deletion of the desaturase and oleate content increased from 62 to 82%.

The resulting strain, *Δsct1 AaSCT1 ole1::PgOLE1Δfad2*, still contains 10% palmitate (C16:0). We attempted to shift this fatty acid into the C18 pool through expression of the rat elongase *rELO2*, an enzyme previously shown to increase oleate content in *S. cerevisiae* [28]. A sucrose utilization cassette was used to express *rELO2* (*Y. lipolytica EXP1* promoter). At the same time, an unmarked cassette for the expression of *Rhodotorula toruloides DGA1* (*Y. lipolytica GPD1* promoter) was co-transformed to increase the overall lipid content of the strain [24]. Transformants were selected on plates containing sucrose as the sole carbon source and then screened for lipid production to identify high lipid content isolates with a high-oleate composition. The highest oleate producer with increased lipid levels was chosen among the *rELO2*-*RtDGA1* transformants.

As a final step to increase oleate content, a phosphite utilization cassette [29] was used to express another copy of *P. graminis OLE1* (*A. adeninivorans TEF1* promoter). A mixture of unmarked cassettes for the expression of *RtDGA1 (Y. lipolytica GPD1* promoter) and *Claviceps purpurea DGA2 (Y. lipolytica TEF1* promoter) were co-transformed with *P. graminis OLE1* to further increase overall lipid content [24]. Transformants were selected on phosphite plates and then screened for lipid production. Strain NS993 emerged as the highest oleate and lipid producer (Fig. 4b, c) reaching 86% oleate and 89% monounsaturated with no polyunsaturated fatty acids in 96-well plate growth conditions.

Random integration transformations yield a range of phenotype expressivity that could be due to different levels of expression from the genomic loci of integration, the number of integration events or to the incidental disruption of an endogenous gene. We resequenced the NS993 genome using PacBio sequencing (Synthetic Genomics, La Jolla CA) to identify the number and sites of heterologous DNA integration. Reads containing matches to the DNA constructs transformed were mapped to the public *Y. lipolytica* genome. We found each gene integrated in one copy per transformation. The only exception was *RtDGA1*, which did not integrate in the final transformation step. The only copy of *RtDGA1* was integrated in tandem with *rELO2*, the result of a single integration during the co-transformation of *RtDGA1* and *rELO2* cassettes in the penultimate step in NS993 engineering (confirmed by PCR on the intermediate strain, data not shown). The copy number of each overexpression cassette was confirmed by qPCR (data not shown). No known open reading frames were disrupted by these integrations. In each case, we found that the construct inserted into a relatively “open” part of a chromosome; the integration site closest to an open reading frame was 477 bp from the 3′ end of a gene, and the closest translation initiation site was 975 bp away. These results indicate that the phenotypes observed are likely explained by optimal expression of a single copy of each construct and point to the location of integration as the major determinant for strength of phenotype.

**Fig. 5:**
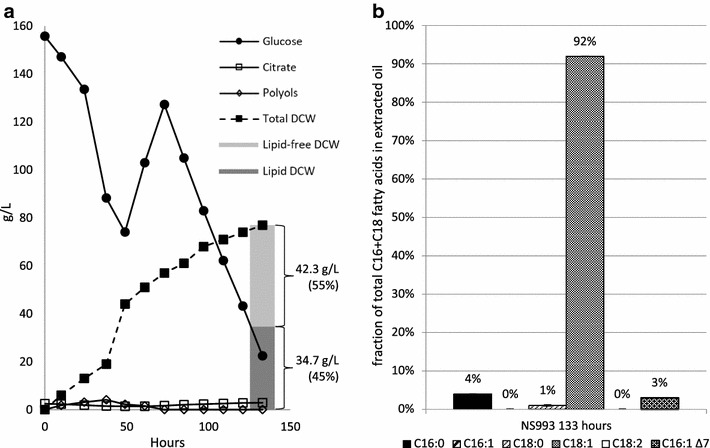
Evaluation of NS993 in a 5L bioreactor and the composition of the resultant lipid. **a** Kinetics of glucose consumption, DCW, citrate, and polyol (summation of erythritol, mannitol, arabitol, and glycerol) production were measured twice daily. The final timepoint was analyzed for lipid content (measured as fatty acid methyl esters), with the C16 and C18 fractions characterized by gas chromatography (**b**)

To assess strain NS993′s growth performance and lipid composition under controlled conditions, a 5-L bioreactor culture was executed. Figure 5a depicts the kinetics of an NS993 glucose fed-batch fermentation through 133 h, the final point in which a sample was taken prior to glucose exhaustion. The culture resulted in minimal citrate and polyol side-product formation and a lipid content, measured by gas chromatography as fatty acid methyl esters (FAME), of 45% of the 77 g/L total yeast dry cell weight (DCW). In comparison with wild-type strain YB-392 as previously reported in Friedlander et al. [24], NS993 exhibited a 1.8-fold increase in lipid content (from 25% in YB-392 to 45% in NS993). It is expected that NS993 lipid content could be further improved to reach levels reported for engineered *Y. lipolytica* strains. Our own previous work led to an engineered strain with 77% lipid content through deletion of the *TGL3* lipase and overexpression of DGATs [24]. Others have shown increased lipid content in *Y. lipolytica* through simultaneous overexpression of *OLE1*, acetyl-CoA carboxylase (*ACC1*), and *DGA1* [30], deletions to affect glycerol-3-phosphate flux and fatty acid degradation [26], as well as an evolutionary approach [31]. NS993 lipid was also characterized for its composition. The same sample used for the lipid content analysis at the end of the fermentation was comprised of 92% oleic acid and 95% monounsaturated fatty acids with no polyunsaturated fatty acids (Fig. 5b).

## Conclusions

We engineered a *Y. lipolytica* strain producing TAG oil with > 90% oleate content and no polyunsaturated fatty acids. The engineered oil surpasses most plant and algal oil in oleate content [32, 33]. Its composition is competitive with oil from experimental Super-high-Oleic (SHO) safflower plants containing 92% oleate [34, 35] and sunflower oil with 89% [36] or up to 94% oleate when precise diurnal temperature control is applied [37]. However, these plant oils contain 1–2.5% [35–38] polyunsaturated fatty acids, whereas NS993 oil has the distinct advantage of completely lacking polyunsaturated fatty acids. The absence of polyunsaturated fatty acids is expected to lead to superior oxidative stability [32]. Industrial production of oil in yeast rather than plants offers flexibility in terms of feedstock, control over production, independence from such variables as weather that affects agricultural crops, and can avoid land use competition with food crops [39, 40]. High-oleate oils have industrial applications including bio-based lubricants, hydraulic fluids, and electrical transformer oils and can serve as a feedstock for the manufacture of oleochemical derivatives including diacids (e.g., azelaic acid), oleyl alcohol, and oleate esters [41, 42]. The strain itself can also form the basis of further engineering for the production of oleic acid derivatives such as ricinoleic acid in vivo [8].

## Methods

### Strains and media

Wild-type *Yarrowia lipolytica* strain YB-392 was obtained from the ARS Culture Collection (NRRL). All strains were cultured in YPD (10 g/L yeast extract, 20 g/L bacto peptone, and 20 g/L glucose) at 30 °C. 20 g/L agar was added to prepare solid media. Antibiotic selection was achieved with the addition of hygromycin B (300 μg/mL), nourseothricin (500 μg/mL), or zeocin (1 mg/mL) as appropriate. Supplementation with 1% Tween 80 and 0.01% oleic acid v/v was used for growth of *ole1::hyg* strains. Sucrose selection was carried out on minimal medium (6.7 g/L Yeast Nitrogen Base without amino acids, 20 g/L agar) supplemented with 20 g/L sucrose. Phosphite selection was carried out on defined medium (5 g/L (NH_4_)_2_SO_4_, 3 g/L K_2_SO_4_, 0.5 g/L MgSO_4_∙7H_2_O, 0.05 mg/L biotin, 25 mg/L myo-inositol, 1 mg/L d-pantothenic acid, 1 mg/L nicotinic acid, 1 mg/L thiamine, 1 mg/L pyridoxine, 0.2 mg/L p-aminobenzoic acid, 1 mg/L H_3_BO_3_, 0.3 mg/L CuSO_4_∙5H_2_O, 0.1 mg/L KI, 0.4 mg/L Na_2_MoO_4_∙2H_2_O, 4.5 mg/L ZnSO_4_∙7H_2_O, 3 mg/L FeSO_4_∙7H_2_O, 1 mg/L MnCl_2_∙2H_2_O, 15 mg/L EDTA, 0.3 mg/L CoCl_2_∙6H_2_O, 4.5 mg/L CaCl_2_∙2H_2_O, 20 g/L glucose, 20 g/L agar) with 1 mM potassium phosphite as the sole phosphorus source [29]. Deep-well plate growth for lipid analysis was performed in a nitrogen-limited medium containing 0.5 g/L urea, 1.5 g/L yeast extract, 0.85 g/L, casamino acids, 1.7 g/L Yeast Nitrogen Base without amino acids and ammonium sulfate, 100 g/L glucose, and 5.1 g/L potassium hydrogen phthalate buffer adjusted to pH 5.5.

### Sequences

The *E. coli hph* gene, conferring hygromycin B resistance (GenBank: AEJ60084.1), the *Streptomyces noursei nat* gene, conferring nourseothricin resistance (GenBank: CAA51674.1), and the *Streptoalloteichus hindustanus ble* gene, conferring zeocin resistance (GenBank: CAA02067.1) were codon-optimized for expression in *Y. lipolytica* (Genscript). *Arxula adeninivorans OLE1* was amplified from genomic DNA prepared from the type strain ATCC 76597. Heterologous *OLE1* genes from *Rhodotorula toruloides*, *Rhodotorula graminis, Microbotryum violaceum*, *Puccinia graminis,* and *Gloeophyllum trabeum* were synthesized (Genscript) without codon optimization based on publicly available sequences: *RtOLE1*-*1* (this gene yielded no viable colonies on unsupplemented media; GenBank: EGU12115.1 [43]), *RtOLE1*-*2* (Fig. 3; GenBank: EMS19868.1 [44]), *RgOLE1* (this gene yielded no viable colonies on unsupplemented media; Joint Genome Institute: 20683), *MvOLE1* (Fig. 3; GenBank: KDE06429.1 [45]), *PgOLE1* (Fig. 3; NCBI Reference Sequence: XP003326562.1) and *GtOLE1* (Fig. 3; and NCBI Reference Sequence: XP 007867726.1 [46]). *Arxula adeninivorans* and *Saccharomyces cerevisiae* GPAT genes were amplified from genomic DNA prepared from the type strains ATCC 76597 and INVSc1 (Invitrogen), respectively. Heterologous GPAT genes from *Rhodotorula toruloides, Rhodotorula graminis* and *Phaeodactylum tricornutum* as well as homologues of the *S. cerevisiae* GPAT gene *GPT2* from *Naumovozyma dairenensis* and *Naumovozyma castellii* were synthesized (Genscript) without codon optimization based on publicly available sequences: *RtSCT1*-*1* (GenBank: EGU12399.1 [43]), *RtSCT1*-*2* (GenBank: EMS25330.1 [44]), *RgSCT1* (Joint Genome Institute: 35710), *PtSCT1* (Joint Genome Institute: 54709), *NdGPT2* (NCBI Reference Sequence: XP_003671489.1 [47]), *NcGPT2* (NCBI Reference Sequence: CCC69529.1 [47]). *Rattus norvegicus* elongase *rELO2* (NCBI Reference Sequence: AC_000070.1 [28]), *Rhodotorula toruloides* DGA1 (GenBank: BAH85840.1 [24]), and *Claviceps purpurea* DGA2 (GenBank: CCE31677.1 [24, 48]) were similarly synthesized without codon optimization based on published sequences. Nucleotide sequences used for heterologous gene expression are provided in the Additional material (Additional file 1).

### Targeted integrations

Targeted genomic integrations were performed as previously described [49]. To delete *Y. lipolytica* genes *SCT1*, *OLE1* and *FAD2*, the appropriate selectable marker gene was amplified by PCR using oligonucleotide primers that attach short flanks homologous to the promoter and terminator of target genes immediately 5′ and 3′ to the ORF in combination with internal marker gene primers. A two-fragment deletion cassette was thus made for each target such that the fragments overlapped in the marker reading frame, but neither fragment alone contained the entire functional antibiotic-resistance gene. For heterologous *OLE1* gene integration into *Ylole1::hph*, the heterologous gene was amplified as a single intact product with flanking regions as described above. All oligonucleotide primers used to amplify deletion cassettes in this study are listed in the additional material (Additional file 2: Tables S1 and S2). PCR products were transformed into hydroxyurea (Sigma-Aldrich)-treated cells [49].

### Gene overexpression

Linear expression constructs were prepared using the standard molecular biology techniques. Each expression construct contained an expression cassette for the gene of interest alone (*RtDGA1*, *CpDGA2*) or in tandem with an expression cassette for a selectable marker (*ble*-*AaSCT1*, *ScSUC2*- *rELO2*, and *ptxD*-*OLE1*). Transformation of these constructs into *Y. lipolytica* strains results in random integration into the genome and can lead to a range of phenotype expressivity. Typically, 90–95 transformants were screened to identify a strain with the most improved lipid profile over the parent strain.

### Transformation

Log-phase *Y. lipolytica* cells were either treated with 50 mM hydroxyurea for 2 h (targeted integration) [49] or were processed directly for transformation (random integration) [24]. Cells were washed with water and resuspended in a volume of water equal to the wet cell pellet. 50 µL was aliquoted per transformation reaction. 18 µL of desired DNA and 92 µL of transformation mix (80 μL 60% PEG4000, 5 µL 2 M DTT, 5 µL 2 M lithium acetate pH 6, and 2 µL 10 mg/mL single stranded salmon sperm DNA) were added to the cell pellet. The transformation reaction was mixed by vortexing and heat shocked at 39 °C for 1 h [50]. Cells were centrifuged, the supernatant was discarded, and cells were resuspended in 1 mL of a suitable non-selective medium, transferred to culture tubes, and cultured overnight at 30 °C before plating on selective media.

### Deep-well plate strain analysis

Strains were inoculated into 300 μL nitrogen-limited media in a 96-well deep-well plate for 72–96 h at 30 °C, 900 rpm and 70–90% humidity (Infors Multitron ATR shaker). To measure fluorescence, cells were first fixed in an equal volume of 100% ethanol at 4 °C for 30 min. 20 μL of the fixed cells were mixed with 80 μL of a master mix containing 0.625 M potassium iodide, 12.5 μM Bodipy 493/503, 0.625% DMSO, and 1.125% PEG 4000 in Costar Black well, clear bottom plates. Fluorescence was measured in arbitrary units with a SpectraMax M2 spectrophotometer (Molecular Devices) at excitation 484 nm and emission 510 nm. The optical density (OD) at 600 nm was measured in the same plate and used to normalize fluorescence readings (FL/OD). To obtain a lipid composition profile, a plate transesterification procedure was developed to extract and convert lipids to FAMEs. Cells were washed with water, pellets were frozen at − 80 °C for 30 min before placing the entire plate in a lyophilizer overnight. Dried cell pellets were crushed in the plate using a 96-pronged metal replicator (Dan-Kar). To each well 500 μL 1.25 M methanol/HCl (Sigma) was added and the plate was sealed closed and incubated at 85 °C for 1.5–2 h with vortexing at 30-min intervals. 1 mL isooctane and 0.5 mL water were then added to each well and mixed by vortexing. A sample of the FAME-containing isooctane layer was analyzed by gas chromatography and composition was determined as percent of total peak area (sum of C16:0, C16:1, C18:0, C18:1, and C18:2) for each FAME species. Because the dry cell weight in each of the 96 wells is not measured, this method yields relative compositional analysis by comparing peak areas within each sample and not quantitative fatty acid levels.

### PacBio strain resequencing

PacBio sequencing (Synthetic Genomics) was carried out on genomic DNA isolated from NS993. Resulting reads were mapped to the sequences of the heterologous DNA constructs used in the creation of NS993 to assemble all reads containing inserted DNA sequence. The resulting 382 reads were mapped against the public *Y. lipolytica* CLIB122 genome to determine the site of each integration.

### Reactors

To the initial 4.4 L batch volume of a fed-batch process executed in a New Brunswick Bioflo 3000 bench-top 5 L (6.6 L total volume) fermentor, medium was added comprising of glucose (150 g/L), (NH_4_)_2_SO_4_ (11 g/L), Sensient Amberex 1003 yeast extract (3 g/L), Sensient Amberferm 4500 corn peptone (0.1 g/L), KH_2_PO_4_ (4 g/L), MgSO_4_·7H_2_O (2 g/L), CaCl_2_·6H_2_O (0.8 g/L), NaCl (0.4 g/L), thiamine * HCl (12 mg/L), biotin (1 mg/L), trace elements [Na_2_MoO_4_·2H_2_O (160 mg/L), CuSO_4_·5H_2_O (0.2 mg/L), H_3_BO_3_ (40 mg/L), MnSO_4_·H_2_O (180 mg/L), FeCl_2_·6H_2_O (75 mg/L), and Antifoam 204 (Sigma-Aldrich) (1 mL/L). Process parameters included an inoculum volume of 2% of the initial batch volume from an overnight shake flask grown in YPD, pH control at 3.5 automatically adjusted with 10 N sodium hydroxide addition, a temperature of 30 °C, aeration at 0.3 vvm air, and agitation controlled between 600 and 1000 rpm to maintain a minimum dissolved oxygen of 20%. A glucose substrate feed was initiated at 37-h post inoculation. A total volume of 1125 mL of a 75% w/v concentrated glucose stock solution was fed at a rate of 32.5 mL/h. A 7-mL fermentation sample was taken twice daily with a Flownamics Seg-flow automated sampler, totaling 84 mL of sampling volume through the 133-h timepoint. Culture supernatants were analyzed by HPLC to determine glucose, citrate, and polyol (mannitol, erythritol, arabitol, and glycerol) concentrations [29]. Furthermore, a 1-mL aliquot of each fermentation sample was washed with an equal volume of water and dried via lyophilization, after which the cell mass was measured to obtain a DCW concentration.

### Lipid extraction and GC analysis of 133-h bioreactor sample

The dried biomass prepared by lyophilization of the 1 mL 133 h fermentation sample was subjected to hexane extraction. The lyophilized cells were bead-beaten for 5 min in a Mini-Beadbeater 8 (Biospec Products) with 425–600-μm glass beads (Sigma) in the presence of hexane (Fisher Chemical). The entire mixture was then added to an oil-less syringe (HSW Norm-Ject) with a 0.22-µM nylon 25 mm disc filter attached. The sample mixture was pushed through the filter into a glass tube and then the filter washed with hexane. All eluate in the tube was blown with compressed air to remove the majority of hexane solvent. The oil was then lyophilized to remove any residual hexane. The weight of this oil represents lipid DCW, while the difference between the initial dried biomass and this value is used to calculate lipid-free DCW. The resultant lipid sample was subjected to transesterification with a solution of 0.5 N HCl in methanol (Sigma) at 85 °C for 2 h. Then, the products (FAMEs) were subjected to a liquid/liquid extraction by the addition of water and then isooctane to the methanol/HCl/products. After vigorously vortexing the two-phase mixture, it was centrifuged to facilitate phase separation. Subsequently, aliquots of the isooctane phase were analyzed with a gas chromatograph (GC; Agilent 7890) equipped with a split/splitless inlet and a flame ionization detector. Separation was achieved with split injection and an Agilent VF-23-MS capillary column (20 m × 0.15 mm × 0.15 µm). Both an internal standard (tridecanoic acid, Sigma) and external standards (Sigma NHI-D FAME Mix) were used for quantification. Percent lipid was reported as the quotient of the sum of the FAMEs (16:0, 16:1Δ7, 16:1Δ9, 18:0, 18:1, 18:2) to the initial yeast DCW.

## Additional files

**Additional file 1.** Heterologous gene sequences used in Tsakraklides et al. 2018.

**Additional file 2: Figure S1.** Confirmation of *FAD2* deletion. 12 transformants and the parental wild-type strain (NS18) screened for the wild-type (top) and deletion (bottom) products. The first isolate was chosen and used for experiments in this study. **Figure S2.** Absence of linoleate in *fad2* strain. A. Gas chromatogram of the parental strain (yellow) and the *fad2::hph* strain (blue). Peaks for the methyl esters of palmitate, palmitoleate, stearate, oleate and linoleate are identified and labeled on the chromatogram by the software based on previously run standards. B. Magnification of the linoleic acid peak for the parental strain (yellow). No detectable linoleic acid is observed for the *fad2::hph* strain (blue). **Table S1.** Primer pairs used to construct targeted integration cassettes. **Table S2.** Primer sequences. **Table S3.** Strains.

## Abbreviations

TAG: triacylglycerol
DNA: deoxyribonucleic acid
qPCR: quantitative polymerase chain reaction
GC: gas chromatography
YPD: yeast extract, peptone, dextrose medium
YNB: yeast nitrogen base medium
FAME: fatty acid methyl ester
OD: optical density
FL/OD: fluorescence divided by optical density
WT: wild-type organism
FAS: fatty acid synthase
GPAT: glycerol-3-phosphate acyltransferase
LPA: lysophosphatidic acid
LPAT: lysophosphatidic acid acyltransferase
PA: phosphatidic acid
PAP: phosphatidate phosphatase
DAG: diacylglycerol
DGAT: diacylglycerol acyltransferase
DCW: dry cell weight
