# Supplementary material for: High-oleate yeast oil without polyunsaturated fatty acids
Source: Biotechnol Biofuels. 2018 May 9;11:131. doi: 10.1186/s13068-018-1131-y (PMC5941336; doi:10.1186/s13068-018-1131-y)
Supplement: Supplementary file 2 — Additional file 2: Figure S1. Confirmation of FAD2 deletion. 12 transformants and the parental wild-type strain (NS18) screened for the wild-type (top) and deletion (bottom) products. The first isolate was chosen and used for experiments in this study. Figure S2. Absence of linoleate in fad2 strain. A. Gas chromatogram of the parental strain (yellow) and the fad2::hph strain (blue). Peaks for the methyl esters of palmitate, palmitoleate, stearate, oleate and linoleate are identified and labeled on the chromatogram by the software based on previously run standards. B. Magnification of the linoleic acid peak for the parental strain (yellow). No detectable linoleic acid is observed for the fad2::hph strain (blue). Table S1. Primer pairs used to construct targeted integration cassettes. Table S2. Primer sequences. Table S3. Strains. [file 13068_2018_1131_MOESM2_ESM.docx]

**Additional file 2**

Confirmation of *FAD2* (*YALIOB10153*) deletion:

Deletion was confirmed by the absence of a *FAD2*-specific PCR product (forward primer NP1888 internal to the *FAD2* gene – reverse primer NP1889 internal to the *FAD2* gene), the presence of a *fad2::hph*-specific PCR product (forward primer NP1890 external to the *FAD2* locus – reverse primer internal to the *hph* gene) (Supplementary Figure 1), as well as the absence of linoleic acid in the resulting strain (Figure 1 and Supplementary Figure 2).

Additional Figure S1


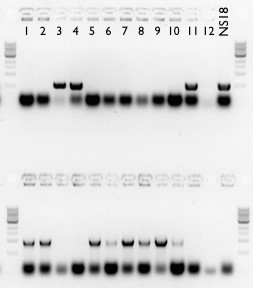


Wild-type *FAD2* product (NP1888-NP1889)

Deletion *fad2::hph* product (NP1890-NP656)

Figure S1. Confirmation of *FAD2* deletion. 12 transformants and the parental wild-type strain (NS18) screened for the wild-type (top) and deletion (bottom) products. The first isolate was chosen and used for experiments in this study

Additional Figure S2


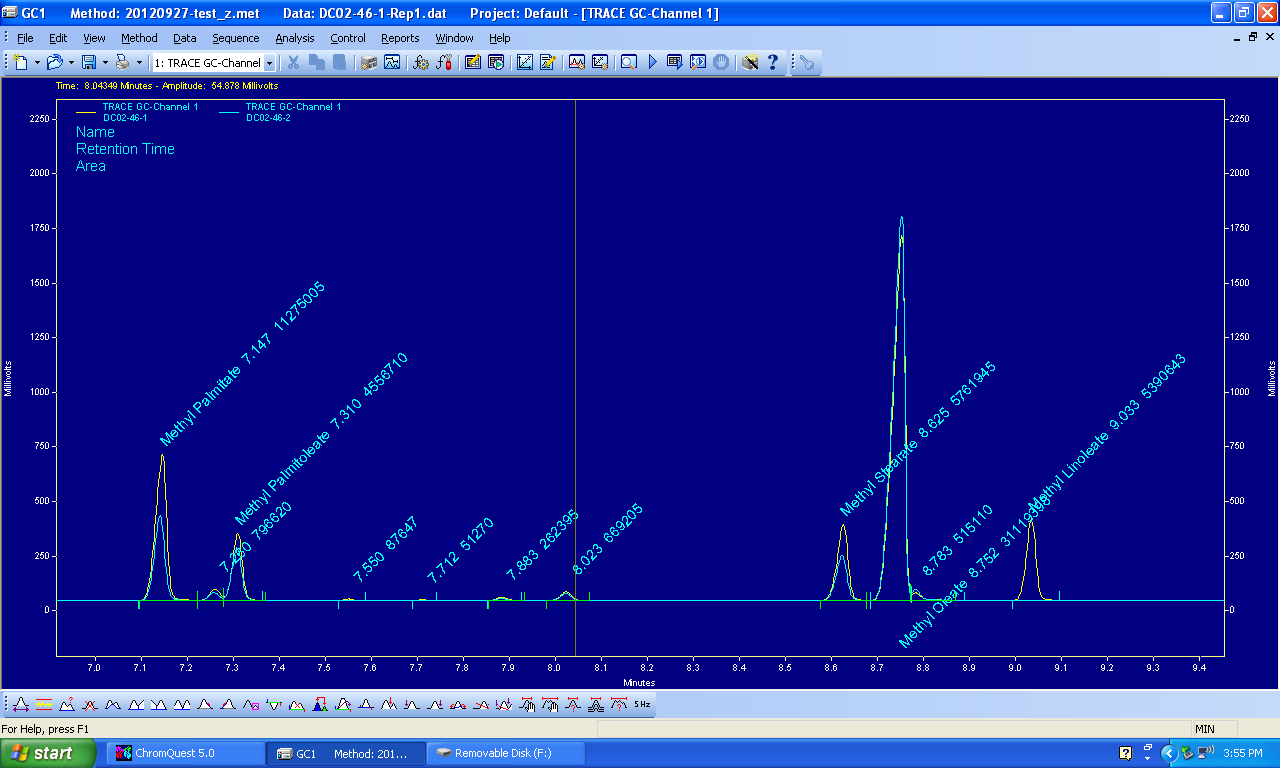
A.


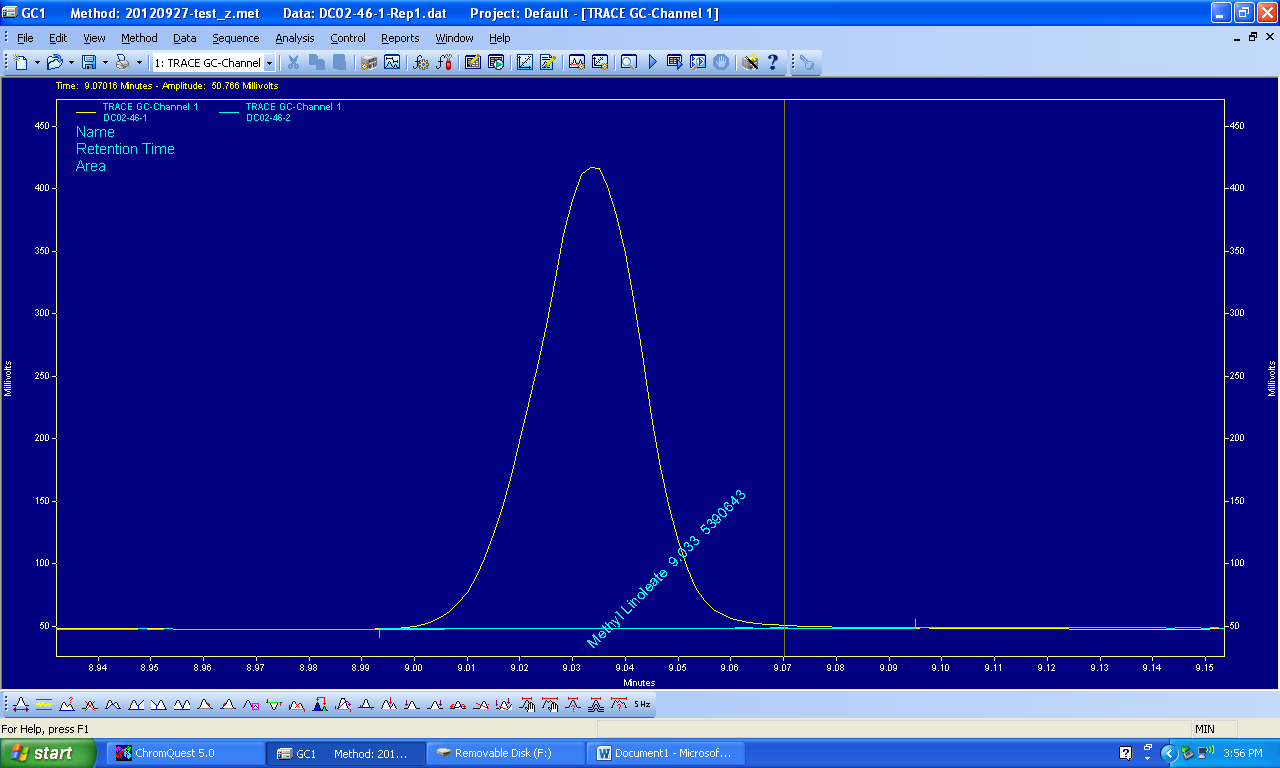
B.

Figure S2. Absence of linoleate in *fad2* strain. A. Gas chromatogram of the parental strain (yellow) and the *fad2::hph* strain (blue). Peaks for the methyl esters of palmitate, palmitoleate, stearate, oleate and linoleate are identified and labeled on the chromatogram by the software based on previously run standards. B. Magnification of the linoleic acid peak for the parental strain (yellow). No detectable linoleic acid is observed for the *fad2::hph* strain (blue).

Table S1. Primer pairs used to construct targeted integration cassettes.

| Targeted modification | 5’ fragment | | 3’ fragment | |
| --- | --- | --- | --- | --- |
|  | forward | reverse | forward | reverse |
| *YlSCT1::nat* | NP2321 | NP356 | NP355 | NP2322 |
| *YlOLE1::hph* | NP1879 | NP656 | NP655 | NP1880 |
| *YlOLE1::AaOLE1* | NP2336 | NP2337 | (single product) | |
| *YlOLE1::RtOLE1-1* | NP2338 | NP2339 | (single product) | |
| *YlOLE1::RtOLE1-2* | NP2340 | NP2341 | (single product) | |
| *YlOLE1::RgOLE1* | NP2342 | NP2343 | (single product) | |
| *YlOLE1::MvOLE1* | NP2344 | NP2345 | (single product) | |
| *YlOLE1::PgOLE1* | NP2346 | NP2347 | (single product) | |
| *YlOLE1::GtOLE1* | NP2348 | NP2349 | (single product) | |
| *Ylfad2::hph* | NP1881 | NP656 | NP655 | NP1882 |

Table S2. Primer sequences.

| NP355 | AACTTCCGTTCCTGGCGATGCCG |
| --- | --- |
| NP356 | TCTCCATCGGAAGCGGTTCCGTC |
| NP655 | ACCACTTGGCGAGACTTCATCTGT |
| NP656 | AGCATCGTCAAAGTTGCCATCCAC |
| NP1879 | GCCGCTTCCCCTACGTAATCGAATACTTGTCTTTCCTCATCTACTAACTACACATTCAACATGAAGAAGCCCGAGCTGACC |
| NP1880 | GGCTCCGATTATTCCTTGTTTCATGCATTATCTATACGATATTAATAAATATTCTTGACTTACTCCTTAGCTCGAGGTC |
| NP1881 | TGAGTCCCTTTCTCGTATTCCAACAGACCGACCATAGAAATGAAGAAGCCCGAGCTGACC |
| NP1882 | TGCTGTTCAGTTAAAACACAATCAAATAACATTTCTAGCTTTACTCCTTAGCTCGAGGTC |
| NP1888 | TCACCGGATGGGTTCTGCAC |
| NP1889 | GGGTGAAGTTCCACTGGTCG |
| NP1890 | TAACCTCGGGTGTGCCTCTG |
| NP2321 | GCGCCACCCTTTGGTTCTTCCAGCACAACAAAAACACGACACGCTAAATGACCACTCTGGATGACACC |
| NP2322 | GCGCACAAAGTCCGTTATAATGAATGATACTCTAAATTTCAACTAGTTAGTTAAGGGCAGGGCATCGACA |
| NP2336 | GCCGCTTCCCCTACGTAATCGAATACTTGTCTTTCCTCATCTACTAACTACACATTCAACATGAACGGTCCCGAAGAGGTG |
| NP2337 | GGCTCCGATTATTCCTTGTTTCATGCATTATCTATACGATATTAATAAATATTCTTGACTTAAGCAGCGCGGCCAGAGA |
| NP2338 | GCCGCTTCCCCTACGTAATCGAATACTTGTCTTTCCTCATCTACTAACTACACATTCAACATGACTGCCTCGTCGGCACT |
| NP2339 | GGCTCCGATTATTCCTTGTTTCATGCATTATCTATACGATATTAATAAATATTCTTGACTTACGCCTTGACCGTCAGGC |
| NP2340 | GCCGCTTCCCCTACGTAATCGAATACTTGTCTTTCCTCATCTACTAACTACACATTCAACATGACTGCCTCTTCGGCACT |
| NP2341 | GGCTCCGATTATTCCTTGTTTCATGCATTATCTATACGATATTAATAAATATTCTTGACTTACGCCTTGACCTTCAGGC |
| NP2342 | GCCGCTTCCCCTACGTAATCGAATACTTGTCTTTCCTCATCTACTAACTACACATTCAACATGCGCCACCCGGACTACTC |
| NP2343 | GGCTCCGATTATTCCTTGTTTCATGCATTATCTATACGATATTAATAAATATTCTTGACTCATGCCGCCCGTGTTGCCG |
| NP2344 | GCCGCTTCCCCTACGTAATCGAATACTTGTCTTTCCTCATCTACTAACTACACATTCAACATGTCCGTCACGGCCTCTGC |
| NP2345 | GGCTCCGATTATTCCTTGTTTCATGCATTATCTATACGATATTAATAAATATTCTTGACCTATTCAAAGAGGGACTGGG |
| NP2346 | GCCGCTTCCCCTACGTAATCGAATACTTGTCTTTCCTCATCTACTAACTACACATTCAACATGTCCAAACCCTCACCTTC |
| NP2347 | GGCTCCGATTATTCCTTGTTTCATGCATTATCTATACGATATTAATAAATATTCTTGACTCAAGTATAGATCGGCTTGACTTC |
| NP2348 | GCCGCTTCCCCTACGTAATCGAATACTTGTCTTTCCTCATCTACTAACTACACATTCAACATGGCGACCTACACGCCGCC |
| NP2349 | GGCTCCGATTATTCCTTGTTTCATGCATTATCTATACGATATTAATAAATATTCTTGACCTACGCTTCCTTCTCCTCCTTC |

Table S3. Strains.

| Strain | Genotype | Source |
| --- | --- | --- |
| NS18 | Wild type | NRRL #YB-392 |
| NS418 | *ole1::hph* | this study |
| NS419 | *fad2::hph* | this study |
| NS432 | *tgl3::hph nat-RtDGA1 ble-CpDGA2* | Friedlander *et al*, 2016 |
| NS563 | *sct1::nat* | this study |
| NS564 | *fad2::hph sct1::nat* | this study |
| NS804 | *sct1::nat ble-AaSCT1* | this study |
| NS808 | *sct1::nat ble-AaSCT1 ole1::hph* | this study |
| NS809 | *sct1::nat ble-AaSCT1 ole1::PgOLE1* | this study |
| NS968 | *sct1::nat ble-AaSCT1 ole1::PgOLE1 fad2::hph* | this study |
| NS975 | *sct1::nat ble-AaSCT1 ole1::PgOLE1 fad2::hph ScSUC2-rELO2 RtDGA1* | this study |
| NS993 | *sct1::nat ble-AaSCT1 ole1::PgOLE1 fad2::hph ScSUC2-rELO2 RtDGA1 ptxD-PgOLE1 CpDGA2* | this study |
